# Contributions of Epstein–Barr Nuclear Antigen 1 (EBNA1) to Cell Immortalization and Survival

**DOI:** 10.3390/v4091537

**Published:** 2012-09-13

**Authors:** Lori Frappier

**Affiliations:** Department of Molecular Genetics, University of Toronto, 1 Kings College Circle, Toronto, ON M5S 1A8, Canada; Email: lori.frappier@utoronto.ca; Tel.: +1-416-946-3501; Fax: +1-416-978-6885

**Keywords:** EBNA1, USP7, PML, p53, survivin, Nm23-H1, ROS, oxidative stress, NFκB, STAT1

## Abstract

Epstein–Barr virus (EBV) immortalizes host cells as part of its latent mode of infection. As a result of this ability to promote cell proliferation and survival, EBV infection contributes to the development of several kinds of B-cell lymphomas and epithelial tumours. The EBV Epstein–Barr nuclear antigen 1 (EBNA1) protein is the only EBV protein expressed in all EBV-associated tumours and plays multiple important roles in EBV latency. In addition to its well-studied roles in viral DNA replication, segregation and transcriptional activation, several studies have identified roles of EBNA1 in manipulating cellular processes that result in reduced apoptosis and increased cell survival. This review discusses these cellular effects of EBNA1 and mechanisms by which they occur.

## 1. Introduction

Epstein–Barr virus (EBV) immortalizes B-lymphocytes as a normal part of its latent infectious cycle. In addition, EBV latent infection is strongly associated with a variety of lymphomas and carcinomas due to its ability to promote the proliferation and survival of these cells. These include Burkitt’s lymphoma, post-transplant lymphomas, Hodgkin’s disease, nasopharyngeal carcinoma (NPC) and gastric carcinoma. There are several forms of EBV latency involving expression of specific subsets of EBV proteins [1,2]. Epstein–Barr nuclear antigen 1 (EBNA1) is the only EBV protein that is expressed in all forms of latency in proliferating cells and in all EBV-associated tumours. It is also the only EBV protein expressed in Burkitt’s lymphoma and in one form of latency. The importance of EBNA1 in EBV infection is reflected in the multiple roles that EBNA1 has been found to play in EBV latency. The first function identified for EBNA1 was in the persistence of latent EBV genomes, which are maintained in the nucleus as circular episomes [3]. EBNA1 was found to be important for the replication and mitotic segregation of the EBV episomes through its direct interaction with sequences in the EBV latent origin of replication (oriP) [4,5,6,7]. EBNA1 can also transactivate the expression of other EBV latency genes through interactions with oriP sequences [8,9]. These functions alone might account for the importance of EBNA1 in latent infection and tumourigenesis in ensuring the persistence and expression of other EBV genes. However, in recent years considerable evidence has accumulated that strongly suggests that EBNA1 also plays a more direct role in cell survival and oncogenesis by altering key cellular proteins and pathways. 

## 2. Evidence for Cellular Effects of EBNA1

Several EBV latent gene products (proteins and small RNAs) contribute to cell proliferation and survival in different forms of EBV latency. This includes EBNA1 which is the only EBV protein expressed in latency I and in some EBV-associated tumours [10,11]. EBNA1 was shown to be important for efficient B-cell immortalization by EBV in tissue culture [12,13]. In addition, several studies have shown that EBNA1 is important for the continued proliferation or survival of some EBV-positive tumour cells. For example, overexpression of EBNA1 mutants, that have a dominant-negative effect on EBNA1 function, decreased cell survival and increased apoptosis in EBV-positive Burkitt’s lymphoma cells but not in EBV-negative B cells [14]. Similarly, EBNA1 silencing in Burkitt’s lymphoma or NPC cell lines decreased cell proliferation [15,16]. While these studies indicate an important contribution of EBNA1 to immortalization, it was not clear whether EBNA1 contributed directly to cell proliferation and survival (*i.e.*, by altering cellular processes) or was simply required to maintain the EBV genomes and expression of other EBV gene products that contribute to these processes.

Other studies have investigated whether EBNA1 expression in the absence of EBV infection is sufficient to affect cell proliferation and survival and to induce tumours. The first evidence that EBNA1 directly affected cell transformation was from a transgenic mouse study in which two mouse lines were generated that expressed EBNA1 at different levels in the B-lymphocytes [17]. Both mouse lines had a high incidence of B-cell lymphomas, and lymphoid cells from these mice were found to have increased growth or survival in culture [17,18]. However, the ability of EBNA1 to induce tumors in transgenic mice is not consistent, as several mouse lines expressing EBNA1 in B-lymphocytes have been generated by another laboratory and were not found to have an increased tumour incidence [19,20]. Similarly these investigators did not detect an effect of EBNA1 expression on the growth or survival of established lymphoblastoid cell lines [21]. Therefore, whether or not EBNA1 expression is sufficient to induce cell transformation is controversial, although it may contribute to oncogenesis under particular conditions. 

The ability of EBNA1 to contribute to the tumourigenicity of EBV-negative cancer cells has been investigated by several groups. EBNA1 expression in HONE-1 NPC cells was found to increase primary tumour formation as well as metastases in nude mice [22]. Similarly, EBNA1 expression in gastric carcinoma cells enhanced tumourigenicity in nude mice [23]. In addition, EBNA1 expression in Hodgkin’s lymphoma cells was found to enhance their ability to form tumours in nonobese diabetic-SCID mice, but not in regular SCID mice [24]. Finally, Kaul *et al.* [25] reported that expression of EBNA1 in a breast carcinoma cell line promoted the rate of tumour growth in nude mice and increased lung metastases. Together the results strongly support a role for EBNA1 in altering cell properties to promote tumour growth.

## 3. Molecular Mechanisms of EBNA1 Cellular Effects

### 3.1. Destabilization of p53

Several aspects of viral infections can induce p53 leading to cell cycle arrest and induction of apoptosis. To avoid this antiviral response, some viral proteins are known to sequester or destabilize p53. While EBNA1 does not bind p53 directly, a connection between EBNA1 and p53 was discovered using proteomics methods (affinity column profiling and tandem affinity purification (TAP) tagging), where EBNA1 was found to interact with the cellular ubiquitin-specific protease USP7 (also called HAUSP [26]). USP7 can bind p53 and Mdm2 (an E3 ubiquitin ligase for p53) and stabilize these proteins by removing the polyubiquitin chains that normally signal degradation [27,28,29]. A combination of biochemical experiments and structural determinations revealed that EBNA1, p53, and Mdm2 compete for the same binding pocket in the N-terminal TRAF domain of USP7, and that EBNA1 uses amino acids 442–448 to contact USP7 [30,31,32,33]. However, EBNA1 binds this pocket with higher affinity than either p53 or Mdm2 and therefore interferes with p53 or Mdm2 binding to USP7 at least *in vitro* [30,31,32]. 

**Figure 1:**
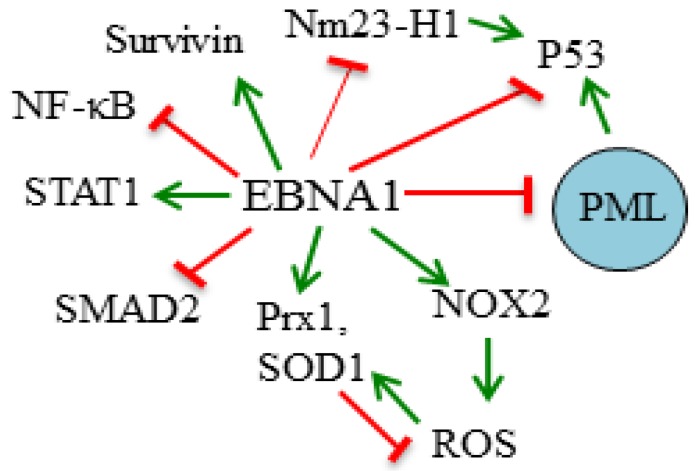
Summary of Epstein–Barr nuclear antigen 1 (EBNA1) effects on cell survival. The cellular proteins whose functions or levels are affected by EBNA1 in ways that likely contribute to increased cell survival are shown. Green arrows represent positive regulation and red blunted lines represent negative regulation. A PML nuclear body is represented by the blue circle.

*In vivo* EBNA1 has been confirmed to lower p53 levels at least in some cell backgrounds (Figure 1). For example, expression of EBNA1 but not a USP7-binding mutant of EBNA1 was shown to reduce the accumulation of p53 in response to DNA damage in U2OS cells [31]. Similarly, EBNA1 expression in CNE2 NPC cells decreased the accumulation of p53 in response to DNA damage [34] and the presence of EBNA1 or EBV in AGS or SCM1 gastric carcinoma cells decreased the steady-state levels of p53 [23,35]. This suggests that EBNA1 is capable of modulating p53 in EBV-infected epithelial cells in ways that could promote cell survival. Accordingly, EBNA1 but not the EBNA1 USP7-binding mutant was shown to decrease DNA damage-induced apoptosis in U2OS [31]. 

### 3.2. Disruption of PML Nuclear Bodies

Promyelocytic leukemia (PML) nuclear bodies (also called ND10s) are nuclear foci based on PML proteins that control several cellular processes including apoptosis, DNA repair and senescence [36,37,38]. Loss of PML proteins or nuclear bodies impairs apoptosis at least in part due to their importance in p53 activation by acetylation [39,40,41]. Accordingly, loss of PML bodies has been associated with the development and/or progression of several tumours [36,42]. In addition, PML proteins are induced as part of the innate antiviral response and suppress productive viral infection in multiple ways [43,44,45]. Several viral proteins have been identified that promote viral infection by disrupting PML nuclear bodies either by interfering with the interactions of PML proteins to form the bodies or by inducing the degradation of the PML proteins [46]. 

Recently EBNA1 was found to induce the loss of PML nuclear bodies in both NPC and gastric carcinoma cells, by promoting the degradation of the PML proteins [34,35] (Figure 1). Downstream effects consistent with PML disruption were also observed, namely decreased abilities to repair DNA damage, acetylate p53 and apoptose in response to DNA damaging agents [34,35]. As mentioned above, EBNA1 also lowers p53 levels in these cells, so the impaired apoptosis in the presence of EBNA1 may be due to a combination of effects. Overall, the data suggest that cells expressing EBNA1 are more likely to survive with DNA damage, which would be expected to contribute to the development of gastric and nasopharyngeal carcinomas, which have increased random DNA damage. Importantly, the EBNA1-induced loss of PML appears to hold up in tumours, as EBV-positive gastric carcinoma tumour samples were found to have considerably less PML than their EBV-negative counterparts [35]. 

The mechanism by which EBNA1 induces the degradation of PML proteins involves EBNA1 binding to both USP7 and the host CK2 kinase and recruitment of these proteins to the PML proteins or nuclear bodies [34,47]. EBNA1 was found to preferentially interact with PML isoform IV over the other five nuclear PML isoforms, and hence the association of EBNA1 with PML IV may target EBNA1 to PML nuclear bodies [34,48]. CK2 has been previously identified as a negative regulator of PML, as phosphorylation of PML proteins by CK2 triggers their polyubiquitylation and degradation [49,50]. EBNA1 binds CK2 directly through amino acids 387–394 which interact with the β regulatory subunit of CK2 [47]. The data suggests that, by increasing the association of CK2 with PML proteins, EBNA1 increases their phosphorylation by CK2 and hence their degradation [47]. Why EBNA1 also requires USP7 to induce PML degradation is less clear. However, additional studies showed that USP7 itself is a negative regulator of PML proteins even in the absence of EBV or EBNA1, and that it induces the degradation of PML proteins independent of its ubiquitin cleavage activity [51]. 

In addition to their cellular roles, PML nuclear bodies have been found to suppress lytic infection by several herpesviruses and this was also recently shown to be true for EBV reactivation to lytic infection [48,52]. Since EBNA1 is expressed in both latent and lytic forms of infection, its ability to degrade PML proteins would be expected to promote EBV lytic infection, and this was recently demonstrated [48]. Together the data suggest that EBNA1’s ability to induce PML loss contributes both to epithelial cancers associated with EBV latent infection and to EBV lytic infection that is known to predominantly occur in the epithelial cells of the orthopharynx. 

### 3.3. Modulation of Signaling Pathways

EBNA1 has been reported to affect several signaling pathways known to regulate cell proliferation and apoptosis (Figure 1). Transcriptional profiling of Ad/AH carcinoma cells with and without stable EBNA1 expression showed that the presence of EBNA1 resulted in increased expression of STAT1, a protein that contributes in multiple ways to apoptotic and non-apoptotic cell death [53,54]. EBNA1 was also found to upregulate STAT1 in HONE-1 NPC and AGS gastric carcinoma cells and to result in enhanced STAT1 phosphorylation and nuclear localization in response to IFNγ [53]. Transcripts that were decreased by EBNA1 include TGF-β1-responsive genes suggesting that EBNA1 also interferes with TGF-β signaling. Subsequent experiments indicated that TGF-β1 signalling was inhibited at least in part because EBNA1 increased the turnover of SMAD2, resulting in decreased levels of SMAD complexes needed for TGF-β1-induced transcription [53]. EBNA1 was subsequently shown to have similar effects on SMAD2 in Hodgkin’s lymphoma cells, resulting in down-regulation of the protein tyrosine phosphatase receptor kappa [55]. However EBNA1 has not been found to physically interact with STAT1 or SMAD2 so it is not clear how EBNA1 elicits these effects.

Another signaling pathway that has been reported to be affected by EBNA1 is the NF-κB pathway. Valentine *et al.* [56] examined the effect of EBNA1 on NF-κB reporter plasmids in carcinoma cell lines and found that EBNA1 inhibited NF-κB activity and DNA binding. Closer examination showed that the levels, nuclear localization and phosphorylation of the p65 NF-κB subunit were all reduced in the presence of EBNA1 as was the phosphorylation of the p65 kinase, IKKα/β. The localization of p65 in NPC biopsies was also found to be cytoplasmic, suggesting that EBNA1 affects NF-κB signalling in the context of NPC. Like the above signaling effects, the effect of EBNA1 on NF-κB seems to be indirect as no physical interaction has been detected between EBNA1 and p65 or IKKα/β. 

### 3.4. Induction of Oxidative Stress

Oxidative stress resulting in the accumulation of reactive oxygen species (ROS) has many cellular effects including induction of apoptosis and DNA damage [57]. EBV infection has been found to be associated with increased oxidative stress [58,59] and this may be at least partly due to EBNA1 expression. Stable or transient EBNA1 expression in B cell lines was found to result in increased levels of ROS, DNA damage foci and dysfunctional, uncapped telomeres, and ROS scavengers were shown to decrease the DNA damage foci and telomere alterations [60,61]. EBNA1 was also found to increase the expression of the NOX2 NADPH oxidase (by an unknown mechanism) which might account for the ROS induction (Figure 1) [60]. Similarly, a comparison of the nuclear proteome in NPC cells with and without EBNA expression showed that EBNA1 upregulated several oxidative stress response proteins including the antioxidants superoxide dismutase 1 (SOD1) and peroxiredoxin 1 (Prx1), that can be induced in response to ROS [62]. Further studies confirmed that ROS levels were elevated by EBNA1 in these cells and that NOX1 and NOX2 transcripts were increased [62]. The combined results suggest that EBNA1 has multiple effects on the oxidative stress response that could affect apoptosis and DNA integrity. 

### 3.5. Inhibition of Nm23-H1

EBNA1 was found to co-immunoprecipitate with Nm23-H1 from lymphoid cells, a known suppressor of metastasis and cell migration, and to relocalize it to the nucleus [63]. This required EBNA1 amino acids 65–89 that are also important in transcriptional activation [63]. Accordingly, EBNA1 was also shown to rescue the suppression of cell migration mediated by Nm23-H1 both *in vitro* and in a nude mouse model, revealing a mechanism by which EBNA1 might contribute to the spread of EBV tumours [25,63]. An independent analysis comparing the nuclear proteomes of NPC cells with and without EBNA1 expression also found that EBNA1 increased the nuclear levels of Nm23-H1, as well as two other proteins involved in metastases (stathmin 1 and maspin), further suggesting that EBNA1 can affect the metastatic potential of cells [62]. 

However the effect of EBNA1 on Nm23-H1 may have additional implications for cell proliferation and survival, as pathway specific microarray analysis recently suggested roles for Nm23-H1 in promoting apoptosis and inhibiting cell proliferation [64] (Figure 1). Specifically, Nm23-H1 overexpression increased some apoptotic transcripts (including caspase3 and 9 and Bcl-X), increased p53 transcripts and decreased cyclin D1 transcripts [64]. Therefore by inhibiting Nm23-H1 function, EBNA1 might decrease apoptosis and increase cell proliferation.

### 3.6. Induction of Survivin

Lu *et al.* [65] used a cell cycle specific array to compare levels of cellular transcripts in EBV-negative B cells with and without EBNA1 expression. They found that EBNA1 increased the levels of survivin transcripts as well as several transcripts associated with cell proliferation. Subsequently, it was shown that EBNA1 associated with the promoter for survivin in B lymphocytes through interactions with the Sp1 host protein, and that this resulted in increased survivin levels. The increase in surviving protein and transcripts required EBNA1 amino acids 65–89, which are known to be important for transcriptional activation of EBV genes [66,67], suggesting that EBNA1 was activating the transcription of the survivin gene. Since survivin inhibits apoptosis, these results suggest that EBNA1 contributes to the survival of EBV-infected cells by increasing survivin expression. 

## 4. Summary

In summary, considerable data suggests that EBNA1 can increase cell proliferation and survival, and may directly contribute to the development of EBV-associated tumours through these effects as well as through the tendency of EBNA1 to increase DNA damage. These effects are likely to involve multiple mechanisms since EBNA1 affects several host proteins and pathways that normally promote apoptosis and regulate cell proliferation. 
